# Complete genome sequence of a broad-host-range lytic *Dickeya* spp. bacteriophage ϕD5

**DOI:** 10.1007/s00705-014-2170-8

**Published:** 2014-07-08

**Authors:** Robert Czajkowski, Zofia Ozymko, Szymon Zwirowski, Ewa Lojkowska

**Affiliations:** 1 Department of Biotechnology, Intercollegiate Faculty of Biotechnology, University of Gdansk, Medical University of Gdansk, Kladki 24, 80-822 Gdansk, Poland; 2 Department of Molecular and Cellular Biology, Intercollegiate Faculty of Biotechnology, University of Gdansk, Medical University of Gdansk, Kladki 24, 80-822 Gdansk, Poland

## Abstract

Little is known about lytic bacteriophages infecting plant-pathogenic *Dickeya* spp. These bacteria cause economically significant losses in arable crops and ornamental plant production worldwide. At present, there is no effective control of diseases caused by *Dickeya* spp. A novel bacteriophage, ϕD5, belonging to the family *Myoviridae*, order *Caudovirales*, that could be used to control these bacteria was isolated previously. This report provides information on its characterization. The ϕD5 genome consists of 155,346-bp-length double-stranded DNA with a GC content of 49.7 % and is predicted to have 196 open reading frames (ORFs) with an average length of 711 nucleotides each. The ORFs were classified into functional groups, including phage structure, packaging, DNA metabolism, regulation, and additional functions. The phage lifestyle predicted from PHACTS indicated that ϕD5 may be a lytic phage and therefore can efficiently kill plant-pathogenic *Dickeya* spp.

**Electronic supplementary material:**

The online version of this article (doi:10.1007/s00705-014-2170-8) contains supplementary material, which is available to authorized users.

*Dickeya* spp. (formerly *Erwinia chrysanthemi* or *Pectobacterium chrysanthemum*) together with *Pectobacterium carotovorum* and *P. atrosepticum* (formerly *Erwinia carotovora* subsp. *carotovora* and *Erwinia carotovora* subsp. *atroseptica*, respectively) are plant-pathogenic bacteria that are responsible for economically important losses in potato and other arable and ornamental crops worldwide [8, 10]. They are recognized to be among the top ten most destructive bacterial pathogens in agriculture [6]. In the past, *Dickeya* spp. were associated mainly with crop diseases in tropical and subtropical regions and were rarely isolated from diseased plants cultivated in Western and Northern Europe [5]. However, recently, there has been an increase in potato crop infection in Europe attributed to *Dickeya* spp., and especially to the presence and spread of a new virulent species named *D. solani* [11]. *D. solani* has been reported in several countries in Western Europe, including France, the Netherlands, Belgium, Poland, Germany, Spain, Sweden, Finland, and the United Kingdom, as well as in Israel and Georgia [11].

At present, there is no effective control of *Dickeya* spp. diseases, whether based on physical, chemical or biological measures under field conditions [3]. There are also no immune crop cultivars readily available on the market. Current control relies solely on measures in crop production that avoid or reduce the risks of contamination of the propagative material – measures that are far from being reliable [3].

Bacteriophages are a promising and environmentally friendly substitute to supplement present control strategies to protect growing plants as well as harvested crops against plant-pathogenic bacteria [7]. Their potential as biological control agents has been evaluated in different studies involving various plant pathogens [6]. Recently, Adriaenssens and co-workers presented a genetic and proteomic study on the first *D. solani* phage, named LIMEstone [1] and we presented data on isolation and characterization of several lytic bacteriophages against different *Dickeya* species [4].

Despite the growing interest in using bacteriophages to control *Dickeya* species, in general, there is still very little information on their genome organization and structure. Data on phage genomics may facilitate development of biological control procedures. For example, the understanding of the genetic background of the phage lifestyle can help in designing better biological control applications, and new delivery strategies may support longer stability of phages in the environment.

So far, there is only one *Dickeya* spp. phage genome available in the GenBank database (NC019925), and this phage has a very restricted host range, able to infect *D. solani* only [1]. In contrast, bacteriophage ϕD5, despite its morphological similarity (both phages belong to the family *Myoviridae*), has a broad host range, larger genome, and higher GC content in comparison with LIMEstone phage [4]. Due to its broad host range, phage ϕD5 appears to be a very good candidate as biological control agent.

The ϕD5 phage was isolated in our previous study from arable soil in the Pomorskie region in Poland, primarily using *D. solani* (strain IPO2222) as a host [4]. It has been characterized in full for its morphologic and phenotypic features. Host-range experiments have showed that it can infect/lyse not only *D. solani* but also other *Dickeya* spp., including *D. dianthicola*, *D. zeae*, *D. dadantii* and *D. chrysanthemi* [4]. Transmission electron microscopy as described by Czajkowski et al. [4] revealed that phage ϕD5 belongs to the family *Myoviridae* and the order *Caudovirales*, with a contractive tail (ca. 140 nm in length) and an icosahedral head (ca. 100 nm in diameter).

To obtain a high phage titer (ca. 10^13^–10^14^ plaque-forming units [pfu] ml^−1^) for genomic DNA purification, phage ϕD5 was enriched in *D. solani* IPO2222 [9] cultures as described previously [2]. After enrichment, bacterial cells were removed by centrifugation (8000×g, 20 min), and the resulting phage suspension (ca. 50 ml) was filtered using a 0.22-µm membrane filter to remove bacterial debris. The phage suspension was treated with DNase I (Sigma-Aldrich; final concentration, 0.5 mg ml^−1^) for 60 min at 37 °C with shaking (100 rpm) to digest the bacterial DNA. Phage particles were further purified and concentrated *via* ultracentrifugation (35,000×g, 4 °C) in a CsCl (Sigma) gradient with densities of 1.4 to 1.7 g ml^−1^ for 3 h following by a second round of equilibrium gradient ultracentrifugation (35,000×g, 4 °C) in 1.4 g ml^−1^ CsCl for 18 h. The resulting high-titer phage suspension (ca. 1.5–2 ml) was dialyzed to remove CsCl at 4 °C in CsCl solutions in water in descending CsCl concentrations (60 % to 10 % CsCl) for 2 h. The ϕD5 phage genomic DNA was isolated as described previously [2].

Phage ϕD5 genomic DNA was sequenced using the Illumina technology and re-assembled *de novo* at Baseclear, The Netherlands (www.baseclear.com). Structural and functional annotation was obtained from the IGS Annotation Service (Institute for Genome Sciences, University of Maryland School of Medicine automated pipeline http://ae.igs.umaryland.edu/cgi/index.cgi) and from RAST (Rapid Annotation using Subsystem Technology, accessed via the internet http://rast.nmpdr.org/). The ϕD5 genome was mapped and annotated using available phage genome sequences deposited in GenBank (http://www.ncbi.nlm.nih.gov/genbank/). The lifestyle of phage ϕD5 (lytic or temperate) was predicted using the PHACTS program (http://www.phantome.org/PHACTS/online.htm).

The genome sequence of phage ϕD5 contains 155,346 bp, 196 predicted open reading frames (ORFs; predicted protein-encoding genes [PEGs]) and an average GC content of 49.7 %. The average gene length was predicted to be 711 nucleotides, and 89.9 % of the genome consists of coding regions. Of the 196 putative PEGs, 50 PEGs (25.5 %) have an assigned function, 42 (21.4 %) were unclassified with no assigned category, and eight (4.1 %) PEGs have unknown function. The rest were classified as (conserved) hypothetical proteins. Functional grouping of predicted ORFs (and PEGs) revealed that two ORFs are associated with nucleotide metabolism, one with energy metabolism, seven with transport and binding proteins, nine with DNA metabolism, three with transcription, one with regulatory functions, four with the cell envelope, six with cellular processes, and four with mobile and extrachromosomal elements (Supplementary Tab. 1). The ϕD5 genome encodes only one tRNA, and for the majority of genes (94.4 %), the start codon for transcription is ATG, whereas GTG and TTG are start codons in only 4.1 % and 1.5 % phage genes, respectively. The functions of the majority of phage ϕD5 genes could not be identified, probably due to the lack of annotation data on phages present in the majority of international genome sequence databases (Fig. 1).

**Fig. 1:**
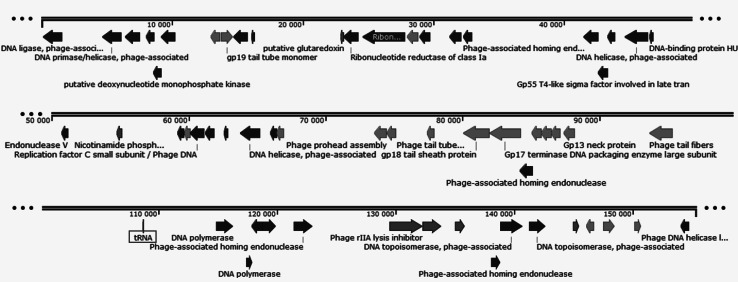
The genome of the bacteriophage ϕD5 (155,346 bp). Structural and functional annotation was obtained from the IGS Annotation Service (Institute for Genome Sciences, University of Maryland School of Medicine automated pipeline http://ae.igs.umaryland.edu/cgi/index.cgi) and from RAST (Rapid Annotation using Subsystem Technology, accessed via the internet http://rast.nmpdr.org/). ORFs coding for proteins involved in DNA/RNA transcription, translation and metabolism are marked in green, ORFs coding for proteins involved in bacteriophage particle assembly are marked in yellow, and ORFs coding for enzymes involved in metabolism are marked in red. The tRNA is marked with a blue square. Arrows indicate the direction of transcription and translation. The ORFs coding for hypothetical and conserved hypothetical proteins are not shown on the map. The figure was generated using the genome visualization program SnapGene ver. 2.3.4 (http://www.snapgene.com/)

To our knowledge, this is the first report of a broad-host-range *Dickeya* spp.-infecting bacteriophage and its complete genome sequence, and it is the first genome sequence of a *Dickeya* spp. bacteriophage isolated in Poland. This sequence will provide useful fundamental information for advanced molecular research on the infection/interaction of phage ϕD5 and its *Dickeya* spp. host.

## Electronic supplementary material

Below is the link to the electronic supplementary material.

Supplementary material 1 (DOCX 25 kb)
