# Supplementary material for: Complete genome sequence of a broad-host-range lytic Dickeya spp. bacteriophage ϕD5
Source: Arch Virol. 2014 Jul 8;159(11):3153–5. doi: 10.1007/s00705-014-2170-8 (PMC4200354; doi:10.1007/s00705-014-2170-8)
Supplement: Supplementary file 1 — Supplementary material 1 (DOCX 25 kb) [file 705_2014_2170_MOESM1_ESM.docx]

**Supplementary table 1.** Summary of the 87 phage φD5 ORFs with predicted, assigned function. ORFs coding for hypothetical proteins and/or coding for conserved hypothetical proteins are not shown in the table.

| **No.** | **Locus taq (ORF)** | **5' end** | **3' end** | **Predicted function (Pfam) ^a^** | **Length (aa) ^b^** | **Taxon** | **% identity** | **P - value** |
| --- | --- | --- | --- | --- | --- | --- | --- | --- |
| 1 | pD5_001 | 1554 | 130 | ATP dependent DNA ligase domain protein | 474 | *Shigella* phage STML-13-1 | 81.4 | 0 |
| 2 | pD5_003 | 3639 | 3355 | NUMOD3 motif family protein | 94 | *Dickeya* sp. phage LIMEStone1 | 99.5 | 6e-67 |
| 3 | pD5_006 | 6111 | 4684 | *dnaB*-like helicase C terminal domain protein | 475 | *Myoviridae* | 99.5 | 1e-77 |
| 4 | pD5_008 | 7509 | 6424 | *recA* bacterial DNA recombination family protein | 361 | Enterobacterial phage T4 | 36.2 | 2e-73 |
| 5 | pD5_010 | 8590 | 8036 | dUTPase family protein | 184 | *Dickeya* sp. phage LIMEStone1 | 93.5 | 1e-127 |
| 6 | pD5_012 | 10199 | 9153 | thymidylate synthase family protein | 348 | *Bacillus* phage SPO1 | 37.7 | 9e-25 |
| 7 | pD5_016 | 13665 | 12964 | putative gp2 DNA end protector protein | 233 | *Dickeya* sp. phage LIMEStone1 | 100 | 1e-177 |
| 8 | pD5_017 | 13720 | 14664 | T4-like virus tail tube gp19 family protein | 314 | *Salmonella* phage SKML-39 | 93.3 | 0 |
| 9 | pD5_018 | 15737 | 14691 | gp32 DNA binding like family protein | 348 | *Enterobacter* phage RB69 | 29.7 | 5e-16 |
| 10 | pD5_020 | 16328 | 16083 | putative gp33 T4-like late promoter transcription accessory protein | 81 | *Dickeya* sp. phage LIMEStone1 | 100 | 4e-48 |
| 11 | pD5_026 | 18550 | 19356 | T4 bacteriophage base plate family protein | 268 | *Dickeya* sp. phage LIMEStone1 | 99.6% | 1e-178 |
| 12 | pD5_028 | 19867 | 21477 | TIGR02594 family protein | 536 | *Salmonella* phage SKML-39 | 94.6 | 0 |
| 13 | pD5_029 | 21550 | 21930 | gene 25-like lysozyme family protein | 126 | *Myoviridae* | 100 | 1e-87 |
| 14 | pD5_032 | 23116 | 22892 | glutaredoxin family protein | 74 | *Dickeya* sp. phage LIMEStone1 | 100 | 7e-46 |
| 15 | pD5_033 | 24229 | 23126 | ribonucleoside-diphosphate reductase 1 subunit beta | 367 | *Enterobacteriaceae* | 46.7 | 3e-95 |
| 16 | pD5_036 | 28825 | 27983 | AAA domain protein | 280 | *Shigella* phage phiSBom-AG3 | 98.9 | 0 |
| 17 | pD5_037 | 29724 | 28930 | putative peptidoglycan binding domain protein | 264 | *Shigella* phage phiSBom-AG3 | 95.5 | 0 |
| 18 | pD5_042 | 32088 | 31228 | putative gp61 DNA primase subunit | 286 | *Dickeya* sp. phage LIMEStone1 | 100 | 0 |
| 19 | pD5_047 | 34836 | 34267 | putative phage associated protein | 189 | *Dickeya* sp. phage LIMEStone1 | 100 | 1e-136 |
| 20 | pD5_048 | 36488 | 34893 | putative phage associated protein | 531 | *Dickeya* sp. phage LIMEStone1 | 99.7% | 0 |
| 21 | pD5_054 | 41472 | 39136 | *recF*/*RecN*/SMC N terminal domain protein | 778 | *Salmonella* phage SFP10 | 71.5 | 0 |
| 22 | pD5_055 | 42590 | 41475 | putative gp47 recombination protein subunit | 371 | *Salmonella* phage SKML-39 | 97 | 0 |
| 23 | pD5_056 | 43318 | 42590 | putative gp55 T4-like sigma factor involved in late transcription | 242 | *Dickeya* sp. phage LIMEStone1 | 100 | 1e-162 |
| 24 | pD5_057 | 43857 | 43330 | RNase H family protein | 175 | *Dickeya* sp. phage LIMEStone1 | 100 | 1e-129 |
| 25 | pD5_058 | 46385 | 44667 | type III restriction enzyme, res subunit | 572 | *Salmonella* phage PhiSH19 | 85.7 | 0 |
| 26 | pD5_059 | 43903 | 44670 | putative conserved phage associated protein | 255 | *Dickeya* sp. phage LIMEStone1 | 85.7 | 0 |
| 27 | pD5_060 | 46793 | 46515 | bacterial DNA-binding family protein | 92 | *Dickeya* sp. phage LIMEStone1 | 100 | 1e-176 |
| 28 | pD5_069 | 51198 | 50743 | endonuclease V | 151 | *Enterobacteriaceae* phage T4 | 42.6 | 9e-15 |
| 29 | pD5_071 | 51945 | 51622 | putative acyl carrier protein | 107 | *Dickeya* sp. phage LIMEStone1 | 100 | 7e-72 |
| 30 | pD5_072 | 54192 | 51988 | von Willebrand factor type A domain protein | 734 | *Klebsiella* phage KP15 | 50.8 | 0 |
| 31 | pD5_074 | 55102 | 54770 | putative nicotinamide phosphoribosyl transferase | 110 | *Dickeya* sp. phage LIMEStone1 | 100 | 3e-72 |
| 32 | pD5_081 | 59654 | 59190 | translation repressor domain protein | 154 | Viruses | 41.3 | 2e-24 |
| 33 | pD5_082 | 60106 | 59684 | putative gp62 clamp loader subunit DNA polymerase accessory protein | 140 | *Dickeya* sp. phage LIMEStone1 | 100 | 1e-98 |
| 34 | pD5_083 | 61100 | 60111 | ATPase associated with various cellular activities family protein | 329 | *Saccharomyces cerevisiae* | 30.8 | 5e-27 |
| 35 | pD5_084 | 61873 | 61205 | gp45 sliding clamp, C terminal family protein | 222 | *Enterobacter* phage RB69 | 25.2 | 2e-08 |
| 36 | pD5_085 | 62713 | 62585 | RNA-DNA and DNA-DNA helicase UvsW domain protein | 42 | *Dickeya* sp. phage LIMEStone1 | 100 | 3e-24 |
| 37 | pD5_088 | 65179 | 63788 | DEAD/DEAH box helicase family protein | 463 | Viruses | 32.1 | 3e-48 |
| 38 | pD5_089 | 65954 | 65208 | PD-(D/E)XK nuclease superfamily protein | 248 | *Dickeya* sp. phage LIMEStone1 | 100 | 0 |
| 39 | pD5_090 | 66409 | 65954 | recombination, repair and ssDNA binding UvsY family protein | 151 | *Dickeya* sp. phage LIMEStone1 | 100 | 1e-107 |
| 40 | pD5_091 | 66952 | 66452 | putative gp3-tail completion | 166 | *Dickeya* sp. phage LIMEStone1 | 100 | 1e-106 |
| 41 | pD5_092 | 68360 | 67632 | putative phage associated protein | 242 | *Dickeya* sp. phage LIMEStone1 | 100 | 1e-142 |
| 42 | pD5_099 | 70483 | 70043 | putative phage associated protein | 146 | *Myoviridae* | 99.3 | 1e-105 |
| 43 | pD5_102 | 72041 | 71193 | NUMOD3 motif family protein | 282 | Viruses | 64.4 | 1e-145 |
| 44 | pD5_103 | 73465 | 72143 | major capsid protein | 440 | *Myoviridae* | 84.1 | 0 |
| 45 | pD5_104 | 74429 | 73557 | putative gp22 prohead core protein | 290 | *Dickeya* sp. phage LIMEStone1 | 100 | 1e-128 |
| 46 | pD5_105 | 75140 | 74475 | prohead core protease family protein | 221 | *Dickeya* sp. phage LIMEStone1 | 100 | 1e-158 |
| 47 | pD5_106 | 75456 | 75151 | putative conserved phage associated protein | 101 | *Dickeya* sp. phage LIMEStone1 | 100 | 1e-53 |
| 48 | pD5_108 | 77364 | 75673 | bacteriophage T4-like capsid assembly family protein | 563 | *Salmonella* phage SKML-39 | 97.3 | 0 |
| 49 | pD5_109 | 77965 | 77432 | tail tube protein | 177 | *Myoviridae* | 87.5 | 1e-105 |
| 50 | pD5_111 | 81971 | 80073 | phage tail sheath family protein | 632 | *Enterobacteriaceae* phage T4 | 32.6 | 1e-42 |
| 51 | pD5_112 | 84231 | 82024 | pretoxin HINT domain protein | 735 | *Enterobacter* phage RB49 | 35.9 | 9e-60 |
| 52 | pD5_113 | 85099 | 84221 | NUMOD3 motif family protein | 292 | *Salmonella* phage SKML-39 | 92.1 | 0 |
| 53 | pD5_114 | 85778 | 85077 | terminase DNA packaging enzyme family protein | 233 | *Dickeya* sp. phage LIMEStone1 | 100 | 1e-151 |
| 54 | pD5_115 | 86476 | 85781 | putative gp15 proximal tail sheath stabilization protein | 231 | *Dickeya* sp. phage LIMEStone1 | 100 | 1e-174 |
| 55 | pD5_116 | 87129 | 86479 | virus neck family protein | 216 | *Myoviridae* | 98.1 | 1e-156 |
| 56 | pD5_118 | 88181 | 87429 | putative gp13 neck protein | 250 | *Salmonella* phage SKML-39 | 95.6 | 1e-162 |
| 57 | pD5_121 | 93630 | 88792 | putative conserved phage associated protein | 1612 | *Salmonella* phage SFP10 | 84.4 | 0 |
| 58 | pD5_122 | 95350 | 93704 | putative tailspike protein | 548 | *Enterobacter* phage KIF | 49.1 | 4e-06 |
| 59 | pD5_123 | 98282 | 95406 | putative tailspike protein | 958 | *Salmonella* phage SKML-39 | 52 | 6e-98 |
| 60 | pD5_125 | 101943 | 98884 | right handed beta helix region family protein | 1019 | *Dickeya* sp. phage LIMEStone1 | 71.6 | 0 |
| 61 | pD5_127 | 104064 | 103210 | putative phage associated protein | 284 | *Salmonella* phage Mo1 | 83.1 | 0 |
| 62 | pD5_128 | 105763 | 104048 | baseplate J-like family protein | 571 | *Enterobacteriaceae* phage T4 | 25 | 4e-25 |
| 63 | pD5_132 | 109550 | 109786 | putative phage associated protein | 78 | *Dickeya* sp. phage LIMEStone1 | 100 | 2e-32 |
| 64 | pD5_133 | 109795 | 110313 | gene 9 domain protein | 172 | *Shigella* phage Sf6 | 43.5 | 2e-18 |
| 65 | pD5_137 | 112768 | 113226 | putative phage associated protein | 152 | *Dickeya* sp. phage LIMEStone1 | 100 | 1e-108 |
| 66 | pD5_141 | 114882 | 116315 | DNA polymerase family B, exonuclease domain protein | 477 | *Enterobacter* phage RB69 | 29.7 | 1e-36 |
| 67 | pD5_144 | 117436 | 117915 | DNA polymerase domain protein | 159 | *Dickeya* sp. phage LIMEStone1 | 98.4 | 1e-112 |
| 68 | pD5_145 | 118791 | 119924 | DNA polymerase B family protein | 377 | *Enterobacter* phage RB69 | 33.7 | 9e-55 |
| 69 | pD5_146 | 119987 | 120328 | putative lipoprotein | 113 | *Dickeya* sp. phage LIMEStone1 | 100 | 2e-77 |
| 70 | pD5_147 | 120325 | 121104 | 5' nucleotidase, deoxy family protein | 259 | *Dickeya* sp. phage LIMEStone1 | 100 | 0 |
| 71 | pD5_150 | 123014 | 123211 | putative membrane protein | 65 | *Dickeya* sp. phage LIMEStone1 | 100 | 2e-39 |
| 72 | pD5_164 | 129512 | 132268 | putative rIIA protein | 918 | Phage vBEcoM | 54.7 | 0 |
| 73 | pD5_165 | 132300 | 133862 | helix-turn-helix domain protein | 520 | *Serratia* phage phiMAM1 | 57.9 | 0 |
| 74 | pD5_168 | 134803 | 135036 | putative orf00005 | 77 | *Dickeya* sp. phage LIMEStone1 | 100 | 2e-50 |
| 75 | pD5_169 | 135015 | 135824 | ig-like virion protein | 269 | *Serratia* phage KSP90 | 45.1 | 3e-06 |
| 76 | pD5_171 | 136149 | 136646 | putative histone-like protein | 165 | *Dickeya* sp. phage LIMEStone1 | 100 | 1e-115 |
| 77 | pD5_172 | 136697 | 136894 | putative membrane protein | 65 | *Dickeya* sp. phage LIMEStone1 | 100 | 4e-39 |
| 78 | pD5_175 | 138058 | 138831 | putative HNH homing endonuclease domain protein | 257 | *Dickeya* sp. phage LIMEStone1 | 100 | 0 |
| 79 | pD5_176 | 138824 | 140722 | histidine kinase-, DNA gyrase B-, and HSP90-like ATPase family protein | 632 | *Drosophila melanogaster* | 35.8 | 7e-92 |
| 80 | pD5_178 | 141300 | 142634 | DNA gyrase/topoisomerase IV, subunit A family protein | 444 | *Schizosaccharomyces pombe* 972h | 32.6 | 4e-53 |
| 81 | pD5_187 | 144982 | 145488 | cytidine and deoxycytidylate deaminase zinc-binding region family protein | 168 | *Dickeya* sp. phage LIMEStone1 | 100 | 1e-118 |
| 82 | pD5_188 | 145499 | 145906 | bacterial PH domain protein | 135 | *Dickeya* sp. phage LIMEStone1 | 100 | 5e-93 |
| 83 | pD5_189 | 146735 | 146118 | putative gp4 head completion protein | 205 | *Dickeya* sp. phage LIMEStone1 | 99 | 0 |
| 84 | pD5_190 | 147524 | 148492 | putative gp48 T4-like baseplate tail tube cap | 322 | Viruses | 94.1 | 0 |
| 85 | pD5_192 | 150183 | 150737 | base plate wedge 53 family protein | 184 | *Dickeya* sp. phage LIMEStone1 | 100 | 1e-133 |
| 86 | pD5_194 | 154742 | 154083 | T4 gene Gp59 loader of gp41 DNA helicase family protein | 219 | *Dickeya* sp. phage LIMEStone1 | 100 | 1e-161 |
| 87 | pD5_195 | 152133 | 154082 | putative phage associated protein | 649 | *Shigella* phage SFP10 | 59.5 | 0 |

^a^ – predicted function is based on amino acid sequence identity, presence of conserved motives, gene location in the functional modules

^b^ - Protein length (number of amino acids)
